# Prognostic value of short-term follow-up BNP in hospitalized patients with heart failure

**DOI:** 10.1186/s12872-017-0632-0

**Published:** 2017-08-03

**Authors:** Sayma Sabrina Khanam, Jung-Woo Son, Jun-Won Lee, Young Jin Youn, Junghan Yoon, Seung-Hwan Lee, Jang-Young Kim, Sung Gyun Ahn, Min-Soo Ahn, Byung-Su Yoo

**Affiliations:** Department of Cardiology, Wonju College of Medicine, Yonsei University, 20 Ilsan-ro, Gangwon-do 26426 Wonju, Republic of Korea

**Keywords:** B-type natriuretic peptide, Heart failure, Prognosis

## Abstract

**Background:**

B-type natriuretic peptide (BNP) has prognostic significance in heart failure (HF), and reductions in BNP may predict clinical improvement. However, there are limited data regarding the prognostic value of BNP during short-term follow-up. The aim of this study was to evaluate the relationship between short-term follow-up BNP and mortality after discharge in patients with HF.

**Methods:**

We analyzed 427 patients hospitalized with HF from the Wonju Severance Christian Hospital Heart Failure Registry from April 2011 to December 2013, with a planned follow-up period through February 2016. Of the 427 patients, 240 (mean age, 75 years; 102 males, 42.5%) had BNP measured on admission and within the short-term follow-up period (3 months). We compared all-cause mortality during the clinical follow-up period (median length of follow-up, 709.5 days) according to the median value of BNP on admission (as a baseline value) and over a short-term follow-up period after discharge.

**Results:**

Median BNP at admission was 816.5 pg/ml, and median follow-up BNP was 369.7 pg/ml. Multivariate analysis revealed a positive association between risk of death and high BNP. High BNP during follow-up was significantly associated with a greater risk of all-cause mortality compared to low BNP (*P* < 0.001). Initial BNP was not significantly associated with all-cause mortality. A multivariate model showed that follow-up BNP and percent change in BNP were independently associated with all-cause mortality after adjustment for covariates. Of the 3 BNP measurement strategies, BNP after discharge (IDI of 0.072, *P* < .0001 and NRI of 0.707, *P* < .0001) and percent change in BNP (IDI of 0.113, *P* < .0001 and NRI of 0.782, *P* < .0001) demonstrated the greatest increase in discrimination and net reclassification for mortality. Unfortunately, we did not find any significant value with initial BNP. Kaplan-Meier survival analysis was performed to assess mortality stratified by BNP according to the median value, high median of follow-up BNP and percent change in BNP were associated with significantly higher mortality compared to the below median (log-rank, *p* < 0.001).

**Conclusions:**

Short-term follow-up BNP and percent change in BNP level are significant prognostic factors of all-cause mortality. These values will be clinically useful when evaluating prognosis in hospitalized patients with heart failure.

## Background

Heart failure (HF) is a global public health concern, especially in countries with aging populations where diagnosis, treatment, and prevention of re-hospitalization are challenging [1]. Unfortunately, repeated hospitalization exerts a huge cost on national healthcare budgets and diminishes quality of life [2]. It is important to predict a patient’s clinical course as early as possible by selecting evidence-based management strategies that improve the care of patients with acute HF. Risk stratification in these patients is challenging [3].The use of natriuretic peptides in the diagnosis of acute HF is well established [4]. Moreover, a peptide that correlates with intra-cardiac pressure is expected to reflect prognosis and predict both short- and long-term outcomes of HF [5]. According to the guidelines of the European Society of Cardiology (ESC) and American College of Cardiology Foundation/American Heart Association (ACCF/AHA), the biomarker B-type natriuretic peptide (BNP) is the most reliable tool for diagnosing HF, establishing prognosis or disease severity, and guiding treatment planning by indicating the need for treatment intensification [6, 7]. Natriuretic peptides such as BNP are counter-regulatory hormones involved in volume homeostasis and cardiovascular remodeling; they have also become promising cardiac markers in various HF settings, especially since the advent of rapid assays [8, 9]. Serial changes in BNP can act as surrogate markers in patients with progressive HF, and these values give incremental prognostic information and help track therapeutic response [10, 11]. A change in BNP during hospitalization appears to predict outcomes in patients with acute decompensated HF (ADHF), providing incremental prognostic value beyond baseline measurement [12]. Also, understanding BNP levels through the early period after discharge may help expedite treatment initiation and allow lower-risk patients to transit more rapidly through the system. However, most studies have evaluated patients only during hospitalization [13, 14]. Variation in BNP level during short-term follow-up is associated with both the degree of congestive status and clinical outcomes [15]. Therefore, this study aimed to evaluate the prognostic value of short-term follow-up BNP and the change in BNP after discharge for predicting mortality of hospitalized patients with HF.

## Methods

### Study population

This study used a subset of data from a larger cohort study, the Korean Heart Failure (KorAHF) registry [16]. We used prospectively collected data for retrospective study analysis. We have only considered same inclusion criteria and registry for sub-set data collection by following the reference. This is the part of Korean HF registry. We performed a single-center cohort study using these data. Patients were diagnosed with acute HF and were admitted to Wonju Severance Christian Hospital (Wonju, Korea).

Data related to in-hospital outcomes and one-year follow-up mortality of 427 consecutive patients who were hospitalized with an episode of acute HF were prospectively collected between April 2011 and December 2013, with a planned follow-up period through February 2016. Data collection, study management, and data validation were performed according to the methods of the KorAHF registry [16]. Study data were obtained for patients with HF if one of the following criteria was met: (i) lung congestion or (ii) findings indicative of LV systolic dysfunction or structural heart disease. Lung congestion was defined as congestion on chest X-ray or as detection of rales on medical examination. Written informed consent was obtained from each patient. If patients were unable to provide consent due to disease severity or other reasons, informed consent was obtained from relatives or a legal guardian. Follow-up data were collected from patients by the attending physician, who completed a web-based case report form. Data were stored in the Clinical Data Management System (iCReaT) under the Korea National Institute of Health (NIH) with the assistance of a clinical research coordinator. In-hospital mortality has been decided by an independent event committee. Mortality data for patients who were lost to follow-up was collected from the National Death Records [16]. The endpoint of the present investigation was defined as all-cause mortality, and the study participants were followed for 730 days from the time they were admitted to the hospital or until death if it occurred earlier. Mortality data were obtained from the National Death Records. All 240 patients underwent follow-up. Variables included demographic and baseline characteristics, medical history, clinical presentation, laboratory tests, hospital course, and clinical outcomes during admission and after one-year of follow-up. We performed echocardiography 1 or 2 days after admission according to the patient’s condition. Initial echocardiographic measurements obtained during index admission were also collected. We evaluated LV end-diastolic volume (LVEDV), LV end-systolic volume (LVESV) and LV ejection fraction (LVEF) parameters using a modified version of Simpson’s method. We did not evaluate echocardiography after 3 months.

### Measurement of BNP

In this study, we selected all the patients that included only BNP level. Plasma BNP level was measured at admission and at the first visit to the outpatient clinic after discharge for short-term follow-up of acute HF. The second blood collection for BNP measurement was performed within 92 days of admission. The median time to second collection was 22 (16 to 33) days. We selected 3 months as the endpoint for the short-term follow-up BNP. All plasma samples were obtained in plastic tubes containing potassium ethylene diamine tetra-acetic acid (Becton Dickinson, Franklin Lakes, NJ, USA) in amounts that ranged from 3 to 5 ml. All samples were centrifuged, and plasma was tested for BNP using the Biosite Triage assay, a point-of-care device that uses a fluorescence immunoassay technique (Biosite, San Diego, CA, USA). The total coefficient of variation at different levels of plasma BNP was reported to be <7% using the control samples provided by the manufacturer. The sensitivity for BNP of these measurements ranged from 5 pg/ml to 5000 pg/ml [17].

### Statistical analysis

Baseline characteristics between the alive and dead group were compared with Mann-Whitney test for continuous variables. Categorical variables are presented as frequency (percentage) and were analyzed via Chi-square test. The prognostic value of the biomarkers was assessed by investigating the relationship with mortality using logistic regression analysis. We categorized the baseline and follow-up biomarker values by median. We used a crude model and models with degrees of adjustment. First, we performed an age- and sex-adjusted analysis (model 1). Second, we adjusted model 1 by including clinical characteristics for hypertension, diabetes, ischemic heart disease chronic obstructive pulmonary disease, chronic kidney disease (CKD), and stroke (model 2). Finally, we adjusted model 2 by including sodium (Na), creatinine (Cr), hemoglobin (Hb) and echocardiographic LVEF (model 3). The increased discriminative value of these biomarkers was examined by calculation of AUC, NRI and IDI. All-cause mortality was estimated with the Kaplan-Meier method and compared using the log-rank test. Statistical significance was set at *p* < 0.05 for all comparisons. All analyses were performed using SAS statistical software (version 9.0; SAS Institute Inc., Cary, NC).

## Results

### Demographic characteristics and clinical profiles

This study enrolled 427 HF patients who had both initial and follow-up BNP data. Patients who died in the hospital and those without follow-up BNP were excluded.

**Fig. 1:**
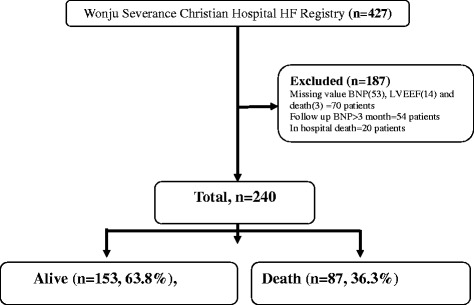
Selection of the study population. This study enrolled 427 patients. We excluded those patients with a creatinine value >2; those who died in-hospital; those for whom the BNP, LVEF, or clinical outcome value (i.e., mortality) was not available; and those for whom the BNP value was measured after more than 3 months. Finally, we analyzed 240 patients, including 153 (63.8%) with alive and 87 (36.3%) with death

We used several exclusion criteria. BNP value is usually increased in CKD patients, so we excluded patients with a creatinine value >2 (43 nos). Those patients who died in hospital after admission were also excluded (20 nos). Those for whom BNP (follow-up = 53 nos), LVEF (14 nos), or clinical outcome data (i.e., mortality) (3 nos) were not available were also excluded. The short-term follow-up period was defined as within 3 months of discharge. Those for whom the BNP value was measured after more than 3 months (54 nos) were not considered (Fig. 1).

The median length of follow-up was 709.5 days (range, 10 to 1477 days). During the follow-up period, 87 (36.3%) patients died. One-year mortality was 47 (19.6%). The median age of the study population was 75 years, and 42.5% of patients were male. Median BNP was 816.5 pg/ml at admission and 369.7 pg/ml on follow-up (median BNP follow-up duration, 22 days).

Baseline characteristics of patients are summarized in Table 1. Age, creatinine level, and the incidence of diabetes (42.5%), hypertension (81.6%), CKD (10.3%), and stroke (25.3%) were significantly higher in patients who died during follow-up compared to those who lived. However, de novo HF (74.5%) was significantly more common among patients who lived than patients who died. In contrast, hemoglobin and sodium were similar between the two groups, and echocardiographic measurements also did not differ significantly.

**Table 1:** Baseline characteristics between the alive and death group

	Alive (*n* = 153)	Death (*n* = 87)	*P* value
Age (years)	73(63 to 80)	78(71 to 82)	<0.001
Males	63 (41.2)	39 (44.8)	0.582
BMI (kg/m2)	22.8(20.5 to 24.8)	22.5(20.2 to 25.5)	0.418
Medical history:			
DM	40 (26.1)	37 (42.5)	0.009
HTN	89 (58.2)	71 (81.6)	<0.001
Congestion	131 (85.6)	72 (82.8)	0.555
De novo HF	114 (74.5)	47 (54)	0.001
COPD	20 (13.1)	17 (19.5)	0.182
CKD	5 (3.3)	9 (10.3)	0.025
IHD	35(22.9)	33(37.9)	0.013
Stroke	16 (10.5)	22 (25.3)	0.002
AF	28 (18.3)	20 (23)	0.383
SBP	140(120.5 to 158)	135(119.5 to 157)	0.352
Echocardiography			
LVESV (mL)	99(57 to 144.5)	82(53 to 137)	0.195
LVEDV (mL)	183(142 to 236.5)	169(129 to 214)	0.072
LVEF (%)	41(32 to 53)	44(32 to 55)	0.388
Laboratory findings			
Hs-CRP (mg/dL)	0.5(0.2 to 2.4)	1.1(0.3 to 2.5)	0.143
Hb (g/dL)	12.8(11.6 to 14.2)	12.8(10.4 to 12.9)	<0.001
Cr (mg/dL)	0.900(0.8 to 1.2)	1.1(0.8 to 1.3)	0.002
Na (mmol/L)	139(137 to 141)	138(135 to 140)	<0.001
Initial BNP (pg/mL)	808.4(497.9 to 1325.3)	872.1(571.3 to 1587.9)	0.165
Follow up BNP (pg/mL)	282.5(136.2 to 487.3)	617.7(319.1 to 1260)	<0.001
(%) Changes of BNP	−66.1(−81.3 to −37.4)	−31.1(−57 to 36.2)	<0.001

Values are expressed as median (25th to 75th) or n (%). *BMI* body mass index, *DM* diabetes mellitus, *HTN* hypertension, *HF* heart failure, *COPD* chronic obstructive pulmonary disease, *CKD* chronic kidney disease, *IHD* ischemic heart disease, left bundle branch block, *AF* atrial fibrillation, *SBP* systolic blood pressure, *LV* left ventricular, *ESV* end-systolic volume, *EDV* end-diastolic volume, *EF* ejection fraction, *Hs-CRP* high-sensitivity C-reactive protein, *Hb* Hemoglobin, *Cr* creatinine, *Na* sodium, *BNP* brain natriuretic peptide

The change in median BNP at admission or initial was not significantly different between the two groups (dead: 872.1 (571.3 to 1587.9) pg/ml; alive: 808.4 (497.9 to 1325.3) pg/ml, *p* = 0.165). However, follow-up BNP was significantly greater in patients who died 617.7 (319.1 to 1260) pg/ml compared to those who lived 282.5 (136.2 to 487.3) pg/ml, *p* < 0.001). Moreover, the median percent change in BNP differed significantly between the two groups (alive: −66.1 (−81.3 to −37.4) %; dead: −31.1 (−57 to 36.2) %, *p* < 0.001) (Table 1).

### Association between BNP and mortality

Logistic regression analysis demonstrated that in the crude model, follow-up BNP and percent change in BNP were significantly associated with mortality. Additionally, when some selective baseline variables (e.g., age, sex, clinical characteristics, Hb, Na and echocardiographic LVEF) were adjusted in the multiple logistic regression model, whereas mortality was the dependent variable; we also found similar results for BNP after discharge and percent change in BNP, which could independently predict primary outcome. However, we did not find any significant association between initial BNP and mortality (Table 2).

**Table 2:** Logistic analysis of BNP at admission, after discharge and (%) changes of BNP (according to the median value) for predicting mortality

BNP	Model		OR (95% CI)	*p*-value
Initial BNP	Crude model	<816.5	1	0.6871
		≥816.5	1.114 (0.658 ~ 1.887)	
	Model 1	<816.5	1	0.4891
		≥816.5	1.215 (0.70 ~ 2.107)	
	Model 2	<816.5	1	0.5791
		≥816.5	1.184 (0.652 ~ 2.152)	
	Model 3	<816.5	1	0.8117
		≥816.5	0.920 (0.465 ~ 1.822)	
Follow up BNP	Crude model	<370	1	<.0001
		≥370	4.30 (2.426 ~ 7.621)	
	Model 1	<370	1	<.0001
		≥370	4.373 (2.406 ~ 7.946)	
	Model 2	<370	1	<.0001
		≥370	4.438 (2.331 ~ 8.450)	
	Model 3	<370	1	<.0001
		≥370	4.703 (2.360 ~ 9.374)	
(%) Changes of BNP	Crude model	<−0.52	1	<.0001
		≥ − 0.52	5.118 (2.852 ~ 9.184)	
	Model 1	<−0.52	1	<.0001
		≥ − 0.52	6.331 (3.353 ~ 11.954)	
	Model 2	<−0.52	1	<.0001
		≥ − 0.52	6.838 (3.418 ~ 13.68)	
	Model 3	<−0.52	1	<.0001
		≥ − 0.52	7.344 (3.518 ~ 15.331)	

Model 1 = Age and sex

Model 2 = Model 1+ diabetes mellitus, hypertension, ischemic heart disease, chronic obstructive pulmonary disease, chronic kidney disease and stroke

Model 3 = Model 2 + sodium (Na), creatinine (Cr), hemoglobin (Hb) and EF

**Fig. 2:**
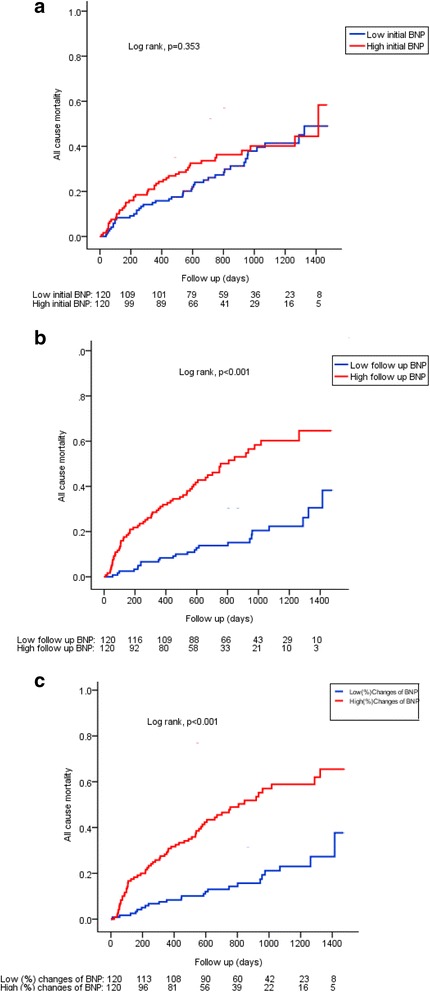
Kaplan-Meier analysis displaying mortality stratified by median value of initial BNP (**a**), follow-up BNP (**b**) and (%) changes of BNP (**c**). High follow up BNP and percent changes of BNP were associated with a higher mortality as compared to low median value (Log-rank, *p* = <0.001)

Kaplan-Meier survival analysis was performed to assess mortality stratified by BNP according to the median value. This analysis revealed that the high median of follow-up BNP and percent change in BNP were associated with significantly higher mortality compared to the below median (log-rank, *p* < 0.001) (Fig. 2).

### Incremental discrimination

The AUC was 0.782 (0.722 ~ 0.842) for the traditional model, which was constructed by using selective baseline variables including age, sex, diabetes mellitus, hypertension, ischemic heart disease, chronic obstructive pulmonary disease, CKD, stroke, Na, Cr, Hb and EF. When we added BNP to the traditional model, we found an AUC of 0.828 (0.777 ~ 0.879) (*P* = 0.0205) for follow-up BNP and 0.852 (0.805 ~ 0.899) (*P* = 0.0023) for percent change in BNP. Furthermore, we compared the discrimination and NRI for each measurement of BNP level in the 240 patients with both admission and discharge BNP data available (Table 3). Of the 3 BNP measurement strategies, BNP after discharge (IDI of 0.072, *P* < .0001 and NRI of 0.707, *P* < .0001) and percent change in BNP (IDI of 0.113, *P* < .0001 and NRI of 0.782, *P* < .0001) provided the greatest increase in discrimination and net reclassification for mortality. There were no significant associations with initial BNP.

**Table 3:** Area under curve (AUC), net reclassification improvement (NRI) and integrated discrimination improvement (IDI).for specific models using the 240 Patients with BNP at admission, after discharge and (%) changes of BNP

BNP		death	
		value	*p*-value
Initial BNP	AUC (95% CI)		
	Traditional model	0.782 (0.722 ~ 0.842)	-
	Traditional model + BNP at admission	0.783 (0.724 ~ 0.843)	0.4683
	NRI (95% CI)	0.014 (−0.25 ~ 0.278)	0.9183
	IDI (95% CI)	0.00013 (−0.002 ~ 0.002)	0.8909
Follow up BNP	AUC (95% CI)		
	Traditional model	0.782 (0.722 ~ 0.842)	-
	Traditional model + BNP at discharge	0.828 (0.777 ~ 0.879)	0.0205
	NRI (95% CI)	0.707 (0.465 ~ 0.949)	<.0001
	IDI (95% CI)	0.072 (0.037 ~ 0.106)	<.0001
(%) Changes of BNP	AUC (95% CI)		
	Traditional model	0.782 (0.722 ~ 0.842)	-
	Traditional model + BNP change	0.852 (0.805 ~ 0.899)	0.0023
	NRI (95% CI)	0.782 (0.544 ~ 1.021)	<.0001
	IDI (95% CI)	0.113 (0.071 ~ 0.155)	<.0001

Traditional model: Age, sex, diabetes mellitus, hypertension, ischemic heart disease, chronic obstructive pulmonary disease, chronic kidney disease, stroke, sodium (Na), creatinine (Cr), hemoglobin (Hb) and EF

## Discussion

We found that both short-term follow-up BNP after discharge and percent change in BNP between admission and follow-up were powerful prognostic markers of mortality in hospitalized patients with HF. In contrast, BNP at admission was not associated with mortality risk. Furthermore, we observed that the high median of follow-up BNP after discharge and percent change in BNP was associated with higher mortality than the below median. This association persisted after adjustment for potential confounders. BNP after discharge and percent change in BNP were independent predictors of mortality.

Many studies have found that BNP level at admission provides useful information for the diagnosis of HF, the assessment of HF severity, and the prognosis of high-risk patients during the early follow-up period [10, 18]. One study that used data from the KorAHF registry found a difference in risk stratification pattern, especially in patients with low NT-proBNP levels [19]. In general, patients with low BNP usually have better prognoses than those with high BNP [19].

Patients hospitalized for HF who are identified as high risk could be scheduled for earlier outpatient visits with a physician and may require follow-up appointments for better outcomes [20]. As expected, BNP concentration 1 month after hospital discharge was the strongest prognostic factor. Another study suggested that HF patients with the highest percentage decrease in BNP 4 months after discharge have the lowest mortality and morbidity [5]. However, no randomized controlled trials have tested whether such data can be used to improve outcomes or have determined the best time for BNP follow-up [18]. In the current study, we found the best cut-off value for monitoring the prognosis of HF to be 294.01 pg/ml for short-term follow-up BNP (within 3 months of discharge; median, 22 days). Median BNP level decreased from 816.5 pg/ml to 369.7 pg/ml (median percent change in BNP, −52.2%) over the 3-month follow-up period. Moreover, short-term follow-up BNP after discharge and percent change in BNP from admission to discharge were associated with mortality. These data may motivate clinicians to measure BNP after discharge to monitor patient outcomes [21]. In Asian countries, including Korea, China, and Japan, the prevalence of HF in the population has increased due to aging of the population and adoption of a Western lifestyle [22]. A few studies have been performed in Asian populations and have revealed that BNP at admission is the most powerful prognostic factor of mortality in patients with HF, and that high levels of BNP are significantly associated with poor outcomes [19, 23]. Most of the studies were designed to investigate the significance of admission BNP for HF patients. Very few studies have explored short-term follow-up BNP. In this study, we mainly focused on Korean HF patients and the effects of short term (90-day) follow-up BNP on prognosis. We also compared the prognostic value of admission BNP, follow-up BNP and percent change in BNP. We measured BNP at admission and at the first follow-up visit to the outpatient clinic after discharge. During the 90-day short term follow-up period after discharge, many patients were lost to follow-up. Thus, unfortunately, our study population is small. However, we analyzed BNP at admission and after discharge and the percent change in BNP and found that the high median for both follow-up BNP and percent change in BNP after short-term follow-up were associated with greater mortality than the below median. We did not find any association between BNP at admission and mortality. After adjustment for covariates of age, sex, clinical characteristics, Hb, Na, and echocardiographic measurement, short-term BNP after discharge and percent change in BNP still predicted mortality. In previous studies, various factors have limited the standard time for evaluating plasma BNP in the prediction of long-term outcomes after discharge [24]. A number of studies have been conducted at single centers or with small sample sizes, limiting the evaluation of the relationship between BNP and other important clinical characteristics; other studies have had variable follow-up times and heterogeneous endpoints [24]. Among those studies that explored the prognostic value of BNP at admission and after discharge, several have also concluded that measurement of BNP at discharge was a strong prognostic marker in HF [25, 26]. One well-designed prospective cohort study of patients with acute HF found that the pre-discharge BNP and percent change in BNP provided conclusive prognostic information and predicted mortality and readmission [27]. Another large, single-center study revealed that high pre-discharge BNP is the most significant predictor of 6-month outcomes and is a more relevant predictor than change in BNP during acute care [2]. However, those studies had small sample sizes, which limited their ability to adjust for covariates [2]. Other studies have found that the percent reduction in natriuretic peptide during HF admission is a powerful predictor of outcome, but the sample sizes were relatively small (less than 200 patients), limiting their ability to control for confounding variables [13, 28]. In the Valsartan Heart Failure Trial (Val-HeFT), BNP was measured at randomization and at the fourth month in patients with chronic HF. The results suggested that percent change in neurohormones such as BNP is associated with mortality and morbidity, thus supporting their role as important surrogate markers in HF [29, 30]. Although there is currently no standard follow-up duration after discharge, our results suggest that short-term follow-up BNP and change in BNP are important metrics for predicting mortality and morbidity in patients with HF. Baseline BNP reflects hemodynamic stress regardless of cause or severity, while follow-up BNP and change in BNP reflect treatment response and hemodynamic status after treatment. Therefore, follow-up BNP and change in BNP are more powerful than baseline BNP for predicting morality or morbidity [18, 31].

HFpEF, defined as symptomatic HF with a normal or almost normal ejection fraction and diastolic dysfunction, accounts for about one-half of patients hospitalized for acute HF [5, 32, 33]. HFpEF patients have comorbidities that can drive myocardial dysfunction and concentric remodeling due to progressive loss of cardiomyocytes with increased relative wall thickness and a relatively preserved LV diameter, resulting in a high ratio of mass to volume. Patients with HFrEF have an enlarged LV cavity but fairly normal LV wall thickness, and exchange of dead cardiomyocytes by collagen creates patchy areas of fibrosis [34, 35]. BNP is a cardiac neurohormone that is primarily exudated from the ventricles in response to increases in wall tension and volume overload [36]. Patients with HFpEF who have lower wall tension thus have lower natriuretic peptide levels than those with HFrEF [37]. The KorAHF registry revealed that the plasma level of natriuretic peptide at baseline or at admission is the most powerful prognostic factor in both HFpEF and HFrEF, and it is also useful for risk-stratifying patients with HF [19]. We used similar inclusion criteria for patients from the same registry; however, we selected whole acute HF patient without any differentiation; interestingly, we found no relationship between BNP at admission and mortality. These differences might be due to the role of baseline BNP in reflecting baseline hemodynamic change, regardless of the cause of HF and severity of underlying disease. Moreover, multiple factors affect the BNP level [18]. Furthermore, when we used new statistical analysis methods (NRI and IDI), we found that BNP after discharge (IDI of 0.072, *P* < .0001 and NRI of 0.707, *P* < .0001) and percent change in BNP (IDI of 0.113, *P* < .0001 and NRI of 0.782, *P* < .0001) demonstrated the greatest increase in discrimination and net reclassification for mortality. This study was limited by a small population. Though the sample size was small but the study was chosen as a cohort study and most of the analytic data set had able to give significance in both FU-BNP and percent changes BNP with little variations. Moreover, a greater decrease in the percent change of BNP from admission to discharge increased the survival rate.

### Study limitations

First, this was a cohort study, so the study design had several inherent limitations, including selection bias and uncontrolled confounding factors. Second, discharge medications and a measure of functional status were not available; therefore, we could not adjust for the use of evidence-based medical therapy or NYHA functional class. Finally, we did not analyze re-hospitalization by HF, so we could not determine whether BNP was a significant predictor of composite mortality and morbidity.

## Conclusion

Short-term follow-up BNP after discharge and percent change in BNP are significant prognostic markers of mortality for hospitalized patients with HF. These values are clinically useful for the evaluation of patient prognosis.

## Abbreviations

AF: Atrial fibrillation
BMI: Body mass index
BNP: Brain natriuretic peptide
CKD: Chronic kidney disease
Cr: Creatinine
DM: Diabetes mellitus
EDV: End-diastolic volume
EF: Ejection fraction
ESV: End-systolic volume
Hb: Hemoglobin
HF: Heart failure
HFpEF: Heart failure with preserved ejection faction
HFrEF: Heart failure with reduced ejection faction
Hs-CRP: High-sensitivity C-reactive protein
HTN: Hypertension
LV: Left ventricular
Na: Sodium
SBP: Systolic blood pressure
